# Impact of the COVID-19 Pandemic on Medical Career Aspirations Among Children of Physicians and Other Healthcare Workers: A Scoping Review

**DOI:** 10.7759/cureus.85994

**Published:** 2025-06-14

**Authors:** Abdousamad Said Omar, Hasan Alchalabi, Amy Gordon

**Affiliations:** 1 General Medicine, Aneurin Bevan University Health Board, Newport, GBR; 2 Surgery, Aneurin Bevan University Health Board, Newport, GBR; 3 Medicine and Surgery, Aneurin Bevan University Health Board, Newport, GBR

**Keywords:** career aspiration, children of physicians, covid-19, healthcare workers, systematic scoping review

## Abstract

Existing reviews examine medical students’ specialty choices and physician‑parent burnout separately; none link parental pandemic experiences to children’s career aspirations. To map and synthesise all empirical evidence on how the COVID‑19 pandemic, particularly parental occupational stress/burnout, has influenced the intentions of children (five to 25 years) of physicians and other healthcare workers (HCWs) to pursue medical careers. We followed the JBI scoping review methodology and PRISMA-ScR guidelines. Databases searched included MEDLINE, Embase, PubMed, ProQuest, and Google Scholar. Screening and data extraction processes involved two reviewers, and quality appraisal was performed using the AXIS checklist. Only one study was eligible, involving 53 adolescents from India. Results indicated nearly equal proportions of participants shifted towards and away from medical careers (15% and 13%, respectively), with the majority (72%) showing no change. There is a substantial gap in the literature regarding this specific impact of the pandemic. Further research, including multi-country and longitudinal studies, is needed.

## Introduction and background

The phenomenon of familial legacy in medicine, where children of healthcare professionals are more likely to pursue careers in the medical field, has been well-documented across various cultures and healthcare systems. In countries like Sweden and Denmark, large-scale cohort studies have revealed a significant increase in the proportion of physicians with at least one physician parent, suggesting a growing intergenerational pipeline into the profession. This trend underscores the profound impact of familial influence on career choices within the medical domain [1].

Several factors contribute to this legacy effect. Children of physicians often have early exposure to the medical environment, access to mentorship, and an intrinsic understanding of the demands and rewards associated with the profession. These elements collectively foster an environment where pursuing a medical career becomes a natural progression. Moreover, the societal prestige and perceived stability associated with medical professions further reinforce this trend [2].

The COVID‑19 pandemic, declared a global health crisis by the World Health Organization, has exerted a profound and multifaceted impact on societies worldwide [3]. Beyond the immediate health implications, the pandemic has triggered significant disruptions across various aspects of life, including the global economy, social interactions, and, most notably, the healthcare system [4]. Healthcare systems globally faced unprecedented strain, characterised by an overwhelming influx of patients, resource limitations, and the urgent need for rapid adaptations in care delivery protocols [3]. This period of intense pressure exposed healthcare workers to considerable physical and psychological risks, fundamentally altering their professional landscape [4-7].

Understanding the comprehensive effects of the pandemic extends to considering its long-term implications for the healthcare workforce, including how it might shape the career aspirations of the next generation of healthcare professionals [3]. The experiences and perceptions of young people, particularly those with close ties to the healthcare system through their parents, are crucial in anticipating future trends in the healthcare workforce supply [3]. Children of physicians and other healthcare workers occupy a unique position during this crisis. They have had a direct and intimate view of the pandemic's impact on their parents' lives, witnessing firsthand the dedication, challenges, and sacrifices associated with healthcare work in such an extraordinary context [8]. This proximity to the realities of the pandemic's frontline could lead to complex and potentially divergent influences on their own considerations of future career paths, especially within the medical field [9].

This scoping review aims to systematically map the available evidence concerning the impact of the COVID‑19 pandemic on the medical career aspirations of children whose parents are physicians or other healthcare workers. By synthesising available literature, this review seeks to provide a comprehensive understanding of the multifaceted effects of the pandemic on this specific population's career interests. The key questions addressed include how the pandemic has influenced these children's interest in pursuing medical careers, the factors driving any observed changes, and the broader implications for healthcare workforce planning.

## Review

Methods

Methodological Framework

The method for this scoping review was based on the framework of Arksey and O’Malley that includes five stages for conducting systemised reviews: (a) identifying the research question; (b) identifying studies that are relevant; (c) selecting studies that meet the inclusion and exclusion criteria; (d) data recording; and (e) collating, summarising, and reporting the results [10].

The review will follow the updated JBI methodology for scoping reviews, and the protocol is written using the JBI scoping review template and the PRISMA Extension for Scoping Reviews (PRISMA‑ScR) checklist. 

The PRISMA-ScR checklist is provided in Appendix A.

Eligibility Criteria

The review question “How has the COVID‑19 pandemic influenced medical‑career aspirations among children of physicians and other HCWs?” was framed with the Population-Concept-Context (PCC) approach (Table 1).

**Table 1:** Population-Concept-Context (PCC) framework along with the inclusion and exclusion criteria for the study

Domain	Inclusion	Exclusion
Participants	Children/adolescents/young adults (five to 25 yrs) with ≥1 physician/HCW parent	General student samples where HCW parent status cannot be isolated
Concept	Impact of COVID‑19‑related parental occupational factors (burnout, workload, infection) on a child’s intention to study medicine	Studies limited to mental health or educational outcomes, with no career measure
Context	Any country; data collected Jan 2020–search date; empirical designs (quant, qual, mixed)	Editorials, opinion pieces, protocols, conference abstracts without full paper, animal studies
Language	English	Non‑English without translation
Types of sources	Peer‑reviewed articles, theses, and technical reports with methods	Blogs, social media posts

Two screening tiers were applied to determine whether articles met the inclusion criteria. Studies had to be peer‑reviewed, published in English between January 1, 2020 and April 26, 2025, and report empirical data (quantitative, qualitative or mixed‑methods). Eligible populations were children, adolescents, or young adults aged five to 25 years who had at least one parent working as a physician or other frontline healthcare worker (HCW). Studies also qualified if they analysed parental HCW sub‑samples separately from a broader cohort. To be included, articles had to examine the impact of the COVID‑19 pandemic, particularly parental occupational exposure, stress, or burnout, on the child’s intention to pursue a medical career. Commentaries, protocols, editorials, conference abstracts without full papers, animal studies and non‑English publications were excluded.

Search Strategy

The research team agreed upon the terms for the search, and one author conducted the searches independently based on those terms before aggregating the sources for all authors’ reviews. Searches across all databases included synonyms and terminological variations with Boolean operators to broaden the results. The initial search evaluated titles and abstracts that included the following terms: “children”, or “adolescents”, or “offspring”, or “teen” and “physician”, or “healthcare personnel”, or “doctor”, or “healthcare worker”, or “clinician” and “COVID-19”, or “SARS-CoV-2”, or “COVID”, or “pandemic” and “career choice”, or “career”, or “vocation”, or “medical school”, or “speciality choice”, or “aspiration”.

The strategies for database search in OVID (Medline and Embase), PubMed, ProQuest, and Google Scholar are provided in Appendix B.

Results

Information Sources

**Figure 1:**
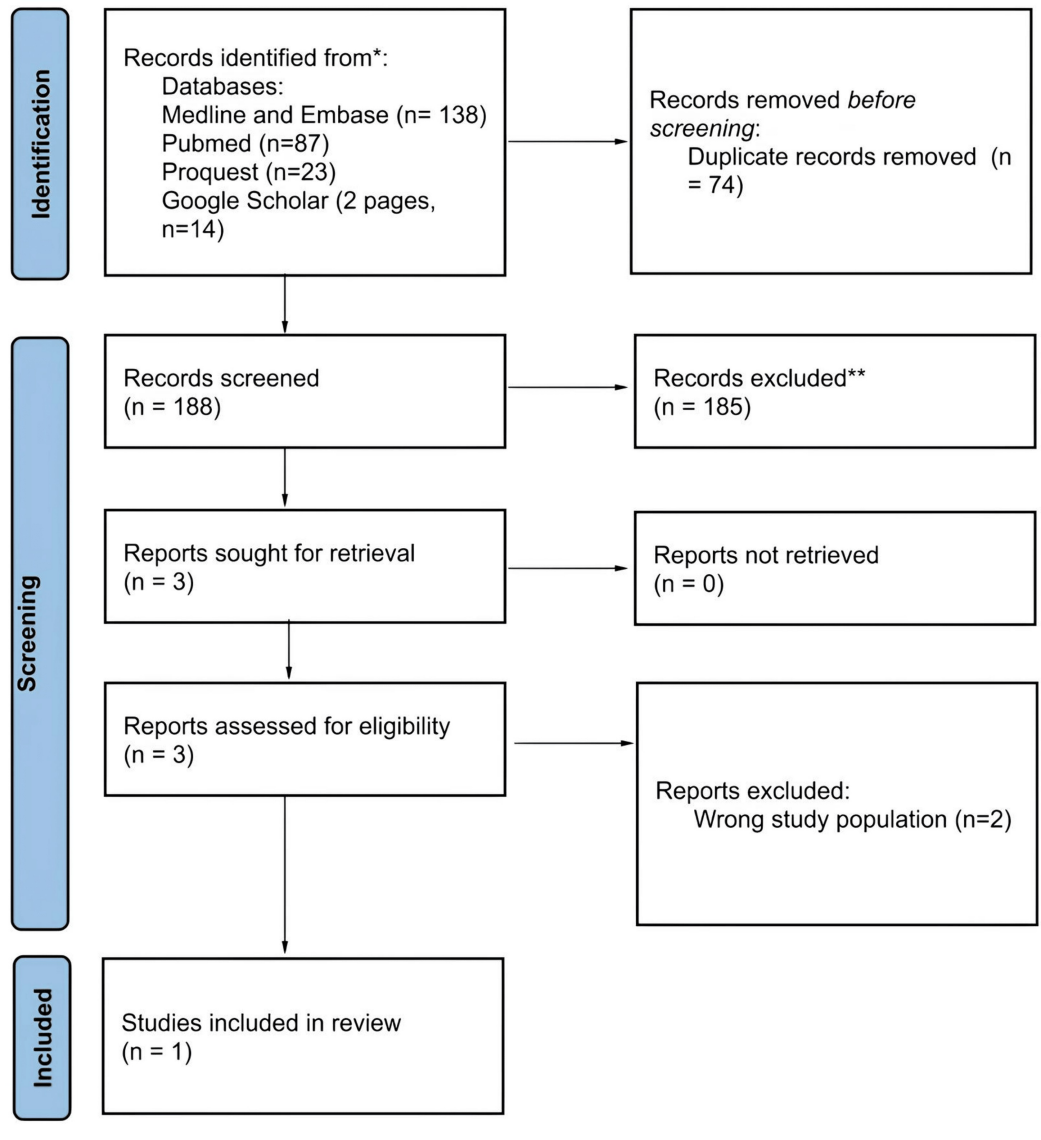
Preferred Reporting Items for Systematic reviews and Meta-Analyses (PRISMA) figure showing the steps to choose the studies for the scoping review

Four electronic sources were searched on April 26, 2025: MEDLINE (Ovid), Embase (Ovid), ProQuest Central (command‑line search across Health & Medicine collections), and Google Scholar (first two result pages). The search returned a total of 262 records, and citations were exported and uploaded to Rayyan.ai, an online screening tool. After removal of duplicates (n = 74), titles and abstracts were screened by one reviewer, yielding three (n = 3) full texts for assessment. A second reviewer resolved any uncertainties. Two articles (n = 2) were excluded at the full‑text stage-as they studied the wrong population, leaving one study for inclusion. Reasons for exclusion were recorded, and the selection process is illustrated in a PRISMA‑ScR flow diagram below (Figure 1).

Data Charting Process

Data extraction was performed in a pilot Excel sheet. One reviewer charted study characteristics and findings; a second reviewer verified all entries. Discrepancies were discussed until consensus was reached, ensuring completeness and accuracy of the single eligible record.

The extraction form captured bibliographic details (author, year, country); study design and setting; sample size and participant age range; parental profession and COVID‑19 exposure variables (infection, frontline workload, burnout measure); description and timing of the career‑aspiration outcome; key quantitative results and qualitative themes; statistical tests used; author‑stated limitations; and reviewer appraisal using the AXIS checklist. These variables were chosen to allow descriptive comparison across designs and to identify evidence gaps by geography, methodology, and outcome measurement.

The article was summarised in a table format based on the study design (Table 2).

**Table 2:** Data extracted of the article in the review

Data extraction	
Reference	Manhata et al. (2022) [9]
Country/setting	India- Urban
Design	Cross-sectional analysis
Data collection format	Online survey
Sample size (n)	54
Age range	10-18 years (mean ≈ 15.1 ± 2.4)
Parental COVID exposure	Front‑line HCWs; 51 % parent infected
Career-aspiration metric	3‑point Likert: likelihood of pursuing medicine (pre‑ and post‑pandemic)
Key findings	A total of 13.2% (n = 7) of participants who initially aspired to medical careers reported a shift toward non-medical fields following the pandemic. Conversely, 15.1% (n = 8) of those previously uninterested in medicine expressed new interest in pursuing it. The majority of participants—71.7% (n = 38)—reported no change in their career intentions. No statistically significant associations were observed between these changes and parental profession, parental COVID-19 infection status, or the child’s age group (all p > 0.05).
Confounder considered	Parent profession; child age group; infection status; death in family/friends
Statistical tests	Fisher’s exact test for categorical comparisons
Limitations	Not mentioned
Reviewer comments	Indian context; school‑based sample; limited relevance to the UK; outcome question single‑item

Critical Appraisal

Although optional for scoping reviews, methodological quality will be appraised to inform gap interpretation: AXIS for quantitative cross‑sectional was used [11]. Scores will not determine inclusion. 

A critical appraisal checklist is provided in Appendix C.

Key Findings

The study investigated the self-reported impact of the COVID-19 pandemic on respondents' career aspirations, particularly the intention to pursue medicine. Prior to the pandemic, participants were almost evenly split in their career preferences between medical and non-medical fields. Following the pandemic, shifts in interest were observed in both directions. A small proportion of respondents reported a transition away from medicine, citing factors such as fear of disease exposure and perceived occupational stress. Conversely, others moved toward considering a medical career, motivated by perceived societal need and altruism.

Despite these directional changes, most respondents remained uncertain or unchanged in their career intentions following the pandemic. Statistical analyses did not reveal any significant associations between career-choice shifts and key demographic or experiential variables such as parental occupation, age group, or direct exposure to COVID-19 through infection or bereavement.

The study also assessed students’ perceptions of health-related risk and professional contribution. While some perceived heightened risk associated with having an HCW parent, the majority viewed the pandemic as a catalyst for healthcare reform and expressed future interest in contributing to the system, either through technology, leadership, or direct service roles.

Methodological appraisal using the AXIS checklist yielded a score of 13 out of 20, indicating moderate quality [11]. Strengths included clearly stated aims, appropriate study design, clearly defined target population, correct use of statistical methods, and comprehensive data presentation. Weaknesses included a lack of sample size justification, absence of response-rate data, no measures for addressing non-responders, and use of an unvalidated single-item measure for career aspiration.

Because only one eligible study was located, quantitative synthesis was not possible. An evidence-gap map shows that data are limited to one developing country, a single cross-sectional design, adolescent participants, and a narrowly defined outcome; no qualitative, longitudinal or high-income-country evidence exists.

Discussion

This scoping review found no empirical research linking the pandemic experiences of physicians and other HCW parents to their children’s aspirations toward medicine. The single Indian observational study suggests a nuanced picture: equal proportions of adolescents moved toward and away from a medical career, while the large majority remained undecided. In other words, first-hand exposure to parental frontline work did not generate either a wholesale exodus from or rush toward the profession [9].

Our finding that Indian HCW offspring did not exhibit a net surge toward medicine contrasts with broader U.S. data: Vasudev et al. surveyed 2,949 high-school students and confirmed that merely having a family member in healthcare was associated with stronger baseline interest in the field (p < 0.0001) [12]. However, that study did not analyse pandemic-driven shifts separately for the HCW-household subgroup, leaving open the question of whether insider stress counterbalanced the "legacy boost".

Similar “outsider” enthusiasm has been documented in China. A nationwide survey of more than 21,000 Chinese senior high-school pupils showed a 12-percentage-point jump in preference for medical study post-outbreak, largely driven by a newfound desire to “contribute to society” [2]. Taken together with Vasudev’s U.S. data, these findings reinforce that the pandemic created powerful prosocial narratives for students without detailed insider exposure [12]. Our lone HCW-offspring study, by contrast, suggests that direct observation of parental stress may blunt - though not reverse - those narratives, yielding a balanced mix of attraction and aversion [9].

Our comparison of the single eligible study focusing specifically on children of HCWs with broader U.S. and Chinese data provides contextual background rather than direct comparability, as these broader studies did not conduct subgroup analyses for HCW offspring specifically [9]. Therefore, any comparisons must be interpreted with caution, and the need for targeted research examining this distinct subgroup is further emphasised.

Several mechanisms could explain the mixed or neutral effect observed in Mahanta et al. Children with direct exposure to parental fatigue, infection risk, and work-family conflict may recalibrate the perceived reward-to-risk ratio of a medical career, offsetting any altruistic pull amplified by media hero narratives [9]. Developmental stage is another factor: intentions at 15 years are notoriously labile; whether the same ambivalence persists into university age is unknown. Cultural context also matters: medicine retains high social prestige in India and China, so deterrent forces must be substantial to counterbalance entrenched regard for the profession, yet the net effect was still neutral [2,13]. Children of HCWs may also experience heightened anxiety due to their parents working in high-risk environments, which could influence their career choices [14].

The evidence base itself is fragile. One moderate-quality, convenience-sample study precludes firm inferences and prevents exploration of publication bias or subgroup effects. Outcome measurement relied on a single unvalidated item, and there was no longitudinal follow-up to test whether attitude shifts endure beyond the acute pandemic phase.

Nevertheless, even this thin evidence raises important questions for workforce planners. Governments that assume the pandemic’s “hero effect” has swollen the future medical-school pipeline may be overlooking a silent counter-current among the very families most embedded in the healthcare culture. Recruitment campaigns might need to address not only altruistic narratives but also concerns about workload, personal risk, and work-life balance conveyed through family role models. The concept of prosocial modelling suggests that while some children might be inspired by their parents' dedication during the pandemic, others might be deterred by witnessing parental stress and burnout [15,16]. The media portrayal of healthcare work during the pandemic, highlighting both the heroic aspects and the immense pressures, could also play a role in shaping these perceptions [17].

Limitations of the Review

This scoping review has several limitations that should be acknowledged. First, the evidence base is notably limited, with only one eligible study identified, substantially restricting the depth, breadth, and generalisability of our conclusions. In addition, the included study was assessed as moderate quality using the AXIS checklist, revealing methodological weaknesses such as a small sample size, use of convenience sampling, lack of sample size justification, and absence of a reported response rate or consideration of non-respondents. Moreover, reliance on a single-item, unvalidated measure for career aspiration limits the reliability and validity of the findings. The decision to include only studies published in English might also have introduced language bias, potentially omitting relevant non-English literature. Furthermore, the primary screening and data extraction were conducted by one reviewer, which may have introduced individual reviewer bias; however, consensus discussions with a second reviewer were used to mitigate this risk. Finally, the search strategy was confined to selected databases and publications available up to April 2025, which means relevant studies indexed elsewhere or published subsequently could have been missed. These limitations underscore the urgent need for more comprehensive, methodologically rigorous, and culturally diverse studies to robustly inform this critical area of research.

Future Research Directions

Future research should focus on addressing the identified gaps and limitations in the current literature. Priorities include (i) multi-country, mixed-method studies that combine validated career-motivation scales with qualitative interviews; (ii) longitudinal cohorts to track career aspiration trajectories as the impact of the pandemic evolves; and (iii) explicit measurement of parental burnout and its mediating effects on their children's career choices. Such research is vital to determine whether the occupational strain faced by HCWs during the COVID-19 pandemic will negatively impact future recruitment into medical careers or potentially inspire a resilient and committed new generation of healthcare professionals.

## Conclusions

In sum, the current literature offers only a single, moderately robust snapshot of how COVID-19 may be shaping the career thinking of HCWs’ children. The snapshot shows neither exodus nor surge, but rather a balance of opposing forces and a large zone of uncertainty. Rigorous, context-specific research is urgently needed to fill this evidence vacuum.

This scoping review will provide the first systematic mapping of how the COVID‑19 pandemic may have influenced medical‑career aspirations among children of HCWs, highlighting evidence gaps and guiding future longitudinal and qualitative studies.

## Figures and Tables

**Figure 2:**
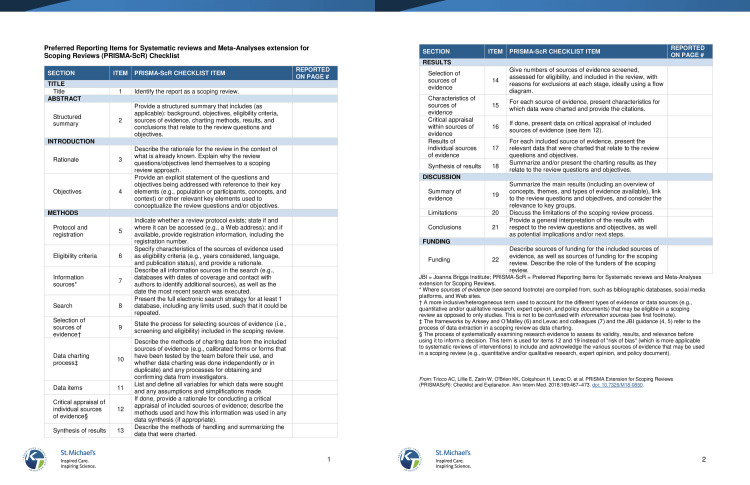
PRISMA for Scoping Reviews (PRISMA-ScR) checklist

**Figure 3:**
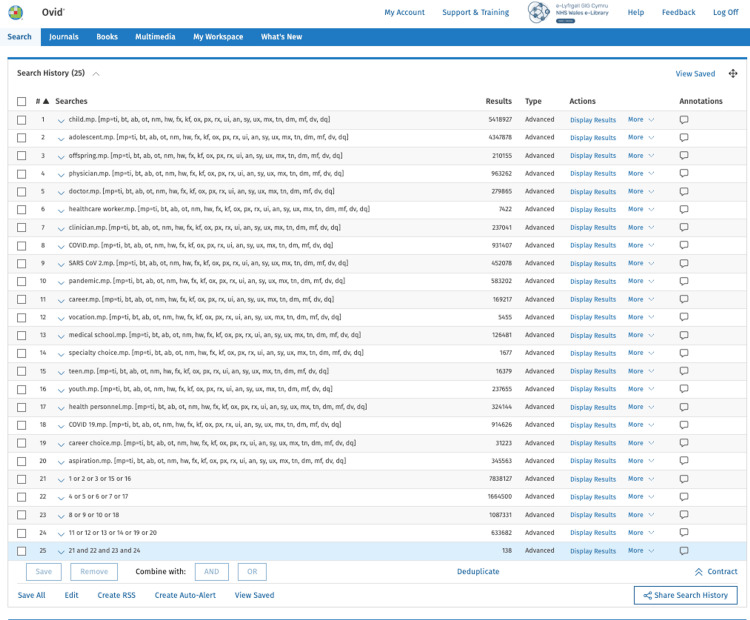
Search strategy for OVID (Medline and Embase)

**Figure 4:**
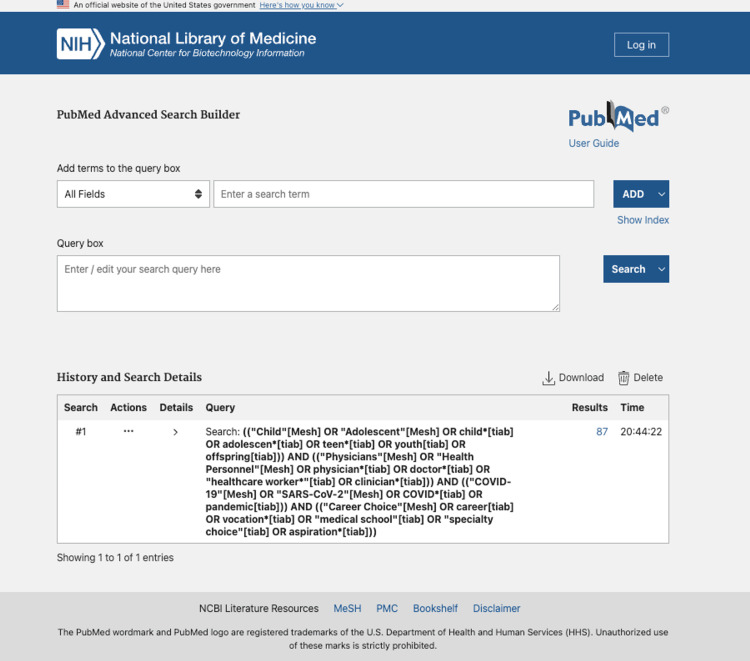
Search strategy for PubMed

**Figure 5:**
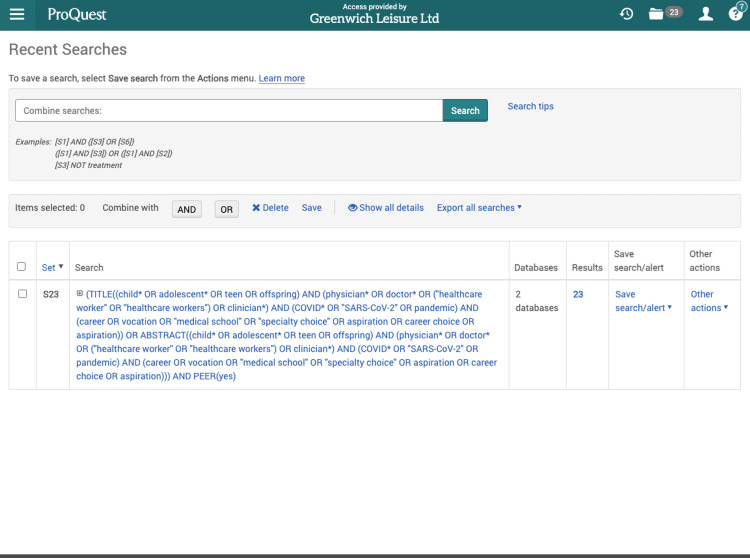
Search strategy for Proquest

**Figure 6:**
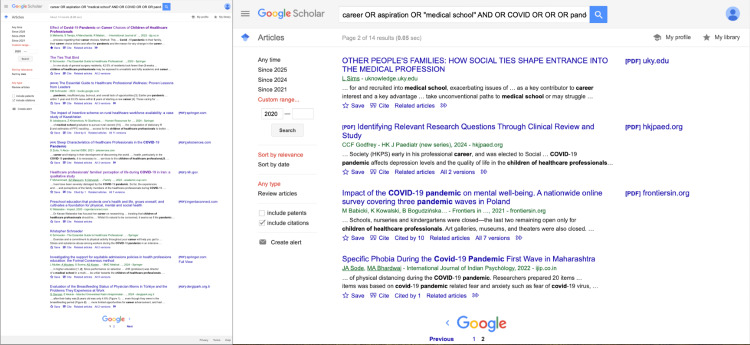
Search strategy for Google Scholar

**Figure 7:**
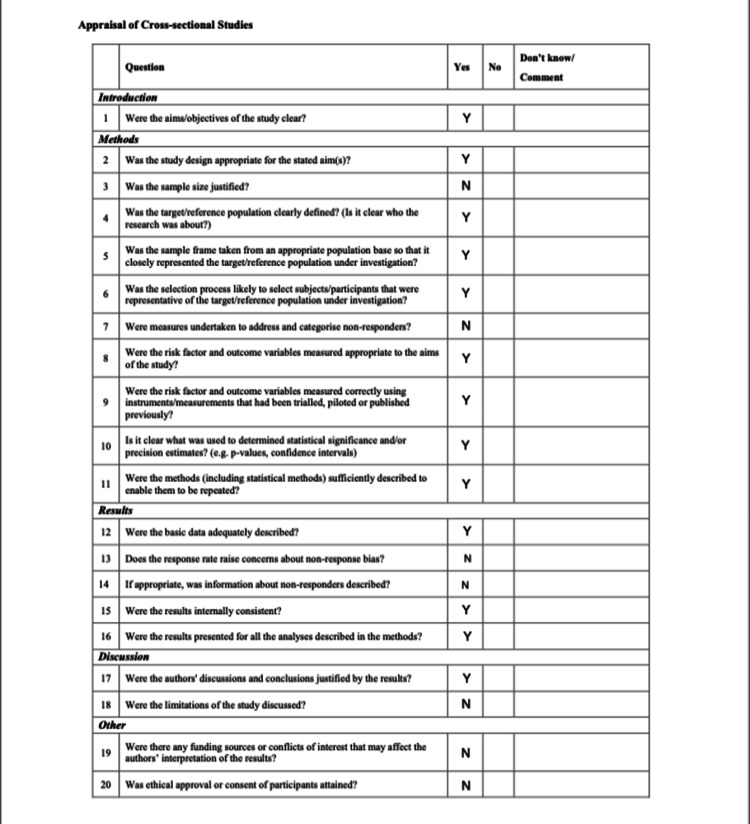
Critical appraisal checklist

## Appendices

Appendix A 

Appendix B 

Appendix C 
